# Human single chain-transbodies that bound to domain-I of non-structural protein 5A (NS5A) of hepatitis C virus

**DOI:** 10.1038/s41598-017-14886-9

**Published:** 2017-11-08

**Authors:** Kittirat Glab-ampai, Monrat Chulanetra, Aijaz Ahmad Malik, Thanate Juntadech, Jeeraphong Thanongsaksrikul, Potjanee Srimanote, Kanyarat Thueng-in, Nitat Sookrung, Pongsri Tongtawe, Wanpen Chaicumpa

**Affiliations:** 1 Graduate Program in Immunology, Department of Immunology, Faculty of Medicine Siriraj Hospital, Mahidol University, Bangkok, Thailand; 2 Center of Research Excellence on Therapeutic Proteins and Antibody Engineering, Department of Parasitology, Faculty of Medicine Siriraj Hospital, Mahidol University, Bangkok, Thailand; 3 Graduate Program in Biomedical Science, Faculty of Allied Health Sciences, Thammasat University, Rangsit Campus, Pathum-thani province 12120 Thailand; 4 School of Pathology, Institute of Medicine, Suranaree University of Technology, Nakhon-ratchaseema province, Thailand

## Abstract

A safe and broadly effective direct acting anti-hepatitis C virus (HCV) agent that can withstand the viral mutation is needed. In this study, human single chain antibody variable fragments (HuscFvs) to conserved non-structural protein-5A (NS5A) of HCV were produced by phage display technology. Recombinant NS5A was used as bait for fishing-out the protein bound-phages from the HuscFv-phage display library. NS5A-bound HuscFvs produced by five phage transfected*-E. coli* clones were linked molecularly to nonaarginine (R9) for making them cell penetrable (become transbodies). The human monoclonal transbodies inhibited HCV replication in the HCVcc infected human hepatic cells and also rescued the cellular antiviral immune response from the viral suppression. Computerized simulation verified by immunoassays indicated that the transbodies used several residues in their multiple complementarity determining regions (CDRs) to form contact interface with many residues of the NS5A domain-I which is important for HCV replication complex formation and RNA binding as well as for interacting with several host proteins for viral immune evasion and regulation of cellular physiology. The human monoclonal transbodies have high potential for testing further as a new ramification of direct acting anti-HCV agent, either alone or in combination with their cognates that target other HCV proteins.

## Introduction

Hepatitis C virus (HCV) is an enveloped plus-sense, single stranded-RNA virus of the genus *Hepacivirus*, family *Flaviviridae*. The virus infects about 3% of the global population leading to more than 700,000 HCV-related deaths annually^1^. Majority of the infection (∼80%) turns to life-long chronic hepatitis which over decades can transform to liver fibrosis, decompensated cirrhosis, and/or hepatocellular carcinoma^2,3^. Currently, there is no effective vaccine against the infection. Before 2011, weekly injected pegylated alpha-interferon (IFNα) together with daily intake of ribavirin (RBV; 1-beta-D-ribofuranosyl-1,2,4-triazole-3-carboxamide) which is a purine analog for 24–48 weeks, often called standard-of-care (SOC) or dual therapy, was a treatment mainstay for HCV infected patients^4^. It is thought that the IFN helps to restore the host innate immunity that has been suppressed by the HCV proteins (NS3/4A protease, NS4B, NS5A and core)^5^ while the RBV causes aberration of the viral replication. Response rates to SOC, defined as sustained virologic response (SVR) or a lack of detectable HCV 12–24 weeks following treatment completion vary greatly depending on the infecting HCV genotypes. Strains of genotypes 1 and 4 are relatively refractory to the regime^6,7^. The SOC protocol is prolonged, highly stringent and also conferred intolerable adverse effects which led to compliance preclusion by a significant portion of the recipients^6^. After 2011, treatment strategy for HCV infection has turned to the use of recently developed direct acting anti-HCV agents (DAAs) which are small molecular inhibitors of the viral enzymes or proteins^8^. The new DAAs together with SOC (triple therapy) or different combinations among the inhibitors themselves conferred improved SVR rates^9–13^. Nevertheless, the novel treatment protocols are too complicate as they must be adjusted according to individual patients’ clinical manifestations, circumstances, and treatment histories as well as the infecting HCV genotypes^13^. They are contraindicated to certain groups of patients^14^ as well as causing additional adverse effects^13^. The new DAA drugs/regimens (particularly when the new inhibitors are administered singly) caused emergence of drug and cross-drug resistant HCV mutants^9,12,13^. Thus, there is still a need of a novel anti-HCV remedy that is safe (preferably interferon-free) and broadly effective against different HCV strains/genotypes.

HCV replication takes place at the virally induced-cellular organelle called replication complex (RC) which is a membrane web on endoplasmic reticulum (ER) of hepatocyte where many HCV and host proteins adjoined^14^. In the infected cell, positive-sense-HCV genome (∼9600 nucleotides) is translated directly into a polyprotein which is cleaved co- and post-translationally by host and viral proteases into four structural (core, E1, E2, and p7) and 6 non-structural (NS) proteins (NS2, NS3, NS4A, NS4B, NS5A, and NS5B) of diverse activities^15^. The NS5A (447 residues) is a hydrophilic multifunctional non-enzymatic protein that pivotally involved in the HCV replication and viral morphogenesis as well as regulation of several cellular signaling pathways^16–19^. The protein modulates hepatic cell physiology for the viral fitness and replication, antagonizes cellular apoptosis and initiates tumorigenicity leading consequently to hepatocellualr carcinoma^16,20–23^. NS5A binds to HCV RNA for replication^24^. The protein exists in two phosphorylated forms, designated p56 and p58 according to their relative molecular masses in electrophoresis (56 and 58 kDa, respectively)^25^. The p56 is basically phosphorylated probably by host casein kinase II at the center and near the C-terminus^26^. The p58 is hyperphosphorylated at the center serine-rich region by cellular kinases, one of which is a casein kinase I-α (CKI-α) with a cooperative mechanism that is believed to involve other HCV NS proteins on the same polyprotein^16,17,27–29^. NS5A N-terminal (residues 5–25) acquires an amphipathic helix (AH) configuration which anchored the protein to the RC web^30,31^. The remaining portion of NS5A in the cytoplasm contains three distinct domains: I (residues 28–213), II (residues 250–342), and III (residues 356–447) which are connected by low complexity sequences (LCS-I and LCS-II)^17,19,32,33^. Domain-I contains a cysteine-rich zinc coordination motif and is essential for HCV replication^17,32^. Crystal structure of the domain-I homodimer revealed a large basic groove at the N-terminalcontact interface of the two molecules; this groove is believed to involve RNA binding during the viral replication^33,34^. Intracellular NS5A regulates cellular physiology and host responses and cooperates with other HCV proteins including NS4B, NS5B and core and also several host proteins for viral replication and assembly^17^. Because the NS5A has multiple pivotal roles in the HCV infectious cycle and pathogenesis, the protein is an attractive target of the newly developed small chemical inhibitors^17,35^.

Usually, mammalian plasma membrane is formidable for hydrophilic peptides and proteins including antibody molecules; thus, conventional antibodies can function only extracellularly^36^. Recently, cell penetrating peptides (CPPs) have been used as vehicles to deliver biologically active, full-length proteins including antibody fragments into living cells^37^. Most CPPs carry positive charge which facilitates electrostatic interactions with negatively charged cell-surface constituents. Typically, they are not exceeding 30 residues in length. Nonaarginine (R9) is one of such CPPs that has been shown to effectively deliver the cargo into cytoplasm^38,39^. In this study, human single chain antibody fragments (HuscFvs) that bound to NS5A were produced by using phage display technology. The HuscFvs were linked molecularly to the R9^39^ in order to make them cell penetrable or become “transbodies”. The R9-HuscFv fusion proteins readily entered HCV infected human hepatic cells, inhibited the viral replication and restored the host innate immunity. Thus, they have potential for further development into another ramification of therapeutic agents against the HCV infection.

## Results and Discussion

### Recombinant full-length NS5A and NS5A domains I (D1), II (D2), and III (D3)

**Figure 1:**
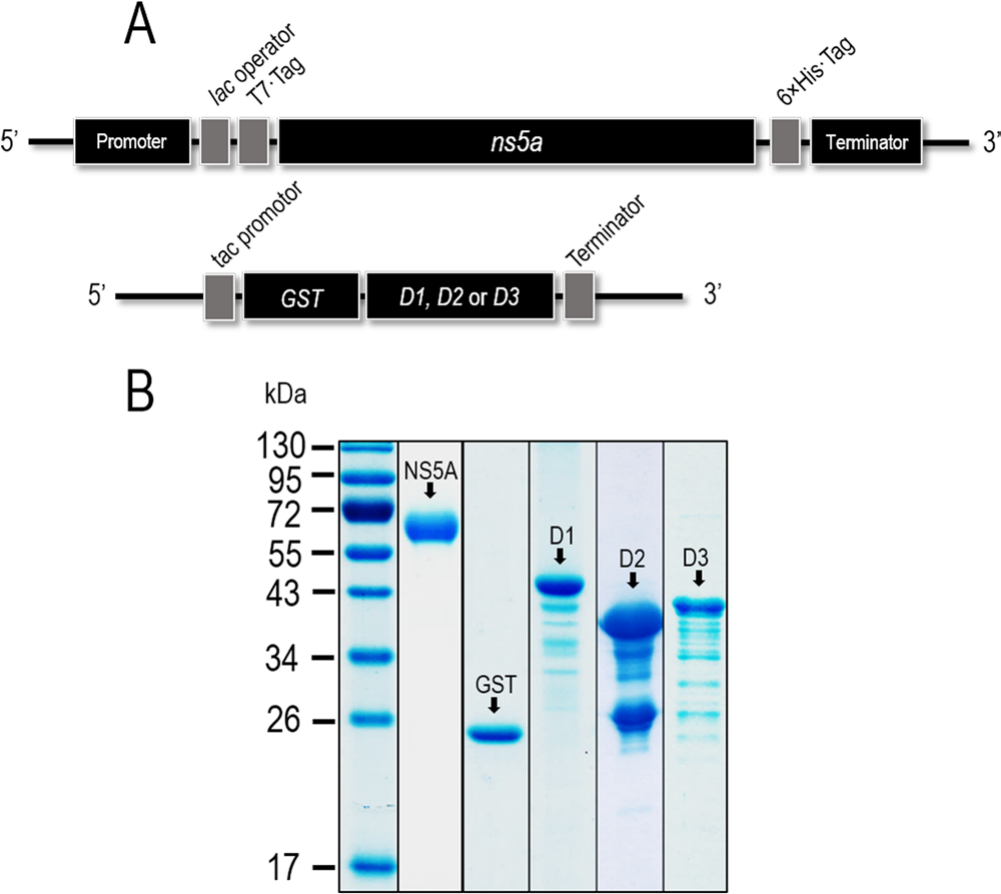
Production of recombinant full-length NS5A protein and domains I (D1), II (D2), and III (D3). Panel A shows schematic representations of the DNA constructs for production of recombinant full length 6× His-tagged-NS5A and glutathione S-transferase (GST)-tagged D1, D2 and D3 of the NS5A. Panel B shows purified recombinant NS5A and D1, D2, and D3. From left to right lanes: PageRuler™ Prestained Protein Ladder, purified 6× His-tagged-NS5A, GST protein, GST-tagged-D1, GST-tagged-D2, and GST-tagged-D3, respectively. Numbers at the left of Panel B are protein molecular masses in kDa.

DNA constructs for production of the recombinant 6× His-tagged-NS5A (rNS5A) and the GST-tagged- D1, D2, and D3 of NS5A in transformed *E. coli* clones carrying the recombinant plasmids with the respective NS5A gene inserts are illustrated in Fig. 1A. The 6× His tag was fused with the recombinant NS5A for facilitating subsequent protein purification by using HisTrap FF column (GE Healthcare, UK) and for tracing the protein by using anti-6× His tag antibody. The relative molecular mass of the rNS5A in the Western blot analysis was about 70 kDa (Fig. 1B). The higher molecular weight of the recombinant protein than the native counterpart (56/58 kDa) should be due to the contiguous 6× His and the additional residues derived from the plasmid flanking regions. The recombinant D1, D2, and D3 of NS5A were produced as GST-tagged proteins and purified by using GSTrap FF affinity column (GE Healthcare) (Fig. 1B). These proteins were used subsequently for mapping the regions of NS5A molecule that were bound by the HuscFvs. All recombinant proteins were verified by LC-MS/MS as the HCV NS5A proteins (data not shown).

### HuscFvs that bound to recombinant NS5A

**Figure 2:**
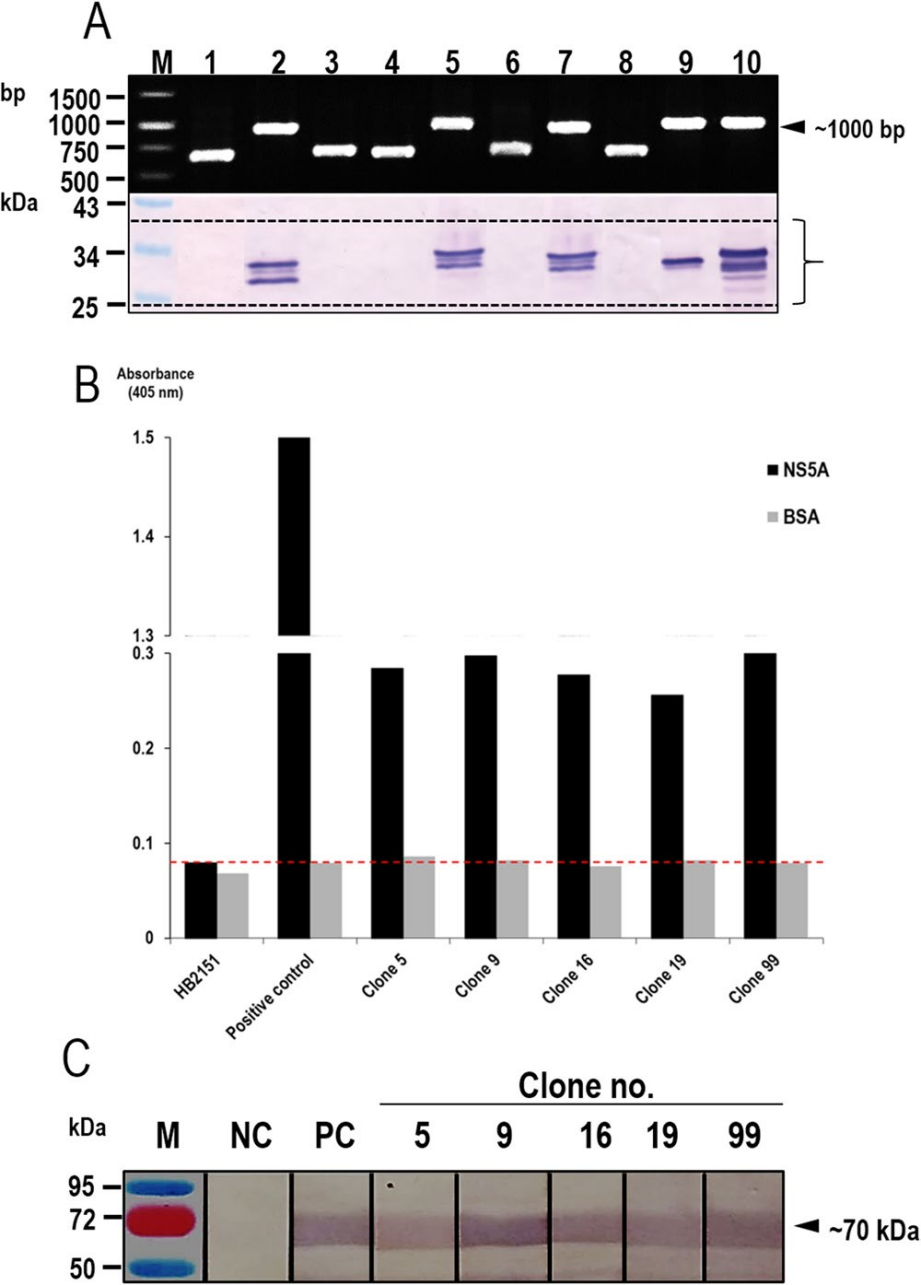
Production of NS5A-bound HuscFvs. Panel A (upper block) shows representative amplicons of HuScFv-coding genes (*huscfvs*) amplified from phage transformed-HB2151 *E. coli* colonies. The molecular mass of the *huscfv* was ∼1,000 bp. Lower block shows HuscFvs produced by representative *huscfv*-positive *E. coli* clones (lanes 2, 5, 7, 9, and 10). Protein doublets are immature HuscFvs with signal peptides (upper bands) and mature HuscFvs without signal peptides (lower bands). Faint bands are degraded products of the principal proteins. Panel B shows results of indirect ELISA (OD_405nm_) for testing binding of the HuscFvs in lysates of the *E. coli* clones 5, 9, 16, 19, and 99 to the HCV NS5A by using BSA as control antigen, lysate of original HB2151 *E. coli* as background antigen-binding control and rNS5A probed with mouse anti-6× His tag as positive control. HuscFvs produced by the five phage transformed-*E. coli* clones gave significant ELISA signals above the controls (dotted line). Panel C shows Western blot results for verification of binding of the HuscFvs to NS5A. The SDS-PAGE-separated NS5A blotted strips were incubated individually with HuscFv5, HuscFv9, HuscFv16, HuscFv19, and HuscFv99; the antigen-antibody reactive bands were revealed by using alkaline phosphatase (AP) conjugated-rabbit anti-E-tag and AP substrate (BCIP/NBT). M is molecular weight marker; NC is negative control which the SDS-PAGE-separated-NS5A blotted strip was incubated with PBS instead of HuscFv; PC is positive control which the SDS-PAGE-separated NS5A blotted strip was probed with mouse anti-6× His antibody, AP-anti-mouse isotype conjugate and BCIP/NBT substrate, respectively.

Full-length rNS5A was used as antigen in the phage biopanning for selecting HuscFv-displayed phage clones from a previously constructed HuscFv-phage display library^39^. The rNS5A-bound phages were used to transfect HB2151 *E. coli* and the bacteria were spread on LB-A selective agar plates. From 300 *E. coli* colonies that grew on the plates, 122 colonies were positive for HuscFv-coding sequences (*huscfvs*) as detected by direct colony PCR^35^. Examples of the *huscfv* amplicons (∼1,000 bp) are shown in upper block of Fig. 2A. Among the 122 *huscfv*-positive HB2151 *E. coli* clones, lysates of 51 clones contained soluble E-tagged-HuscFv proteins after growing the bacteria under IPTG induction condition. Western blot patterns of the HuscFv representatives probed with rabbit anti-E-tag antibody are shown in lower block of Fig. 2A. Among the 51 clones, HuscFvs in lysates of 5 transformed *E. coli c*lones (5, 9, 16, 19, and 99) gave significant indirect ELISA signals (OD_405nm_) to the rNS5A above the BSA control (Fig. 2B). Binding of the HuscFvs of these *E. coli* clones to rNS5A was verified by Western blot analysis (Fig. 2C). NS5A-bound HuscFvs of these *E. coli* clones were used further.

### Cell penetrable monoclonal HuscFvs (transbodies)

**Figure 3:**
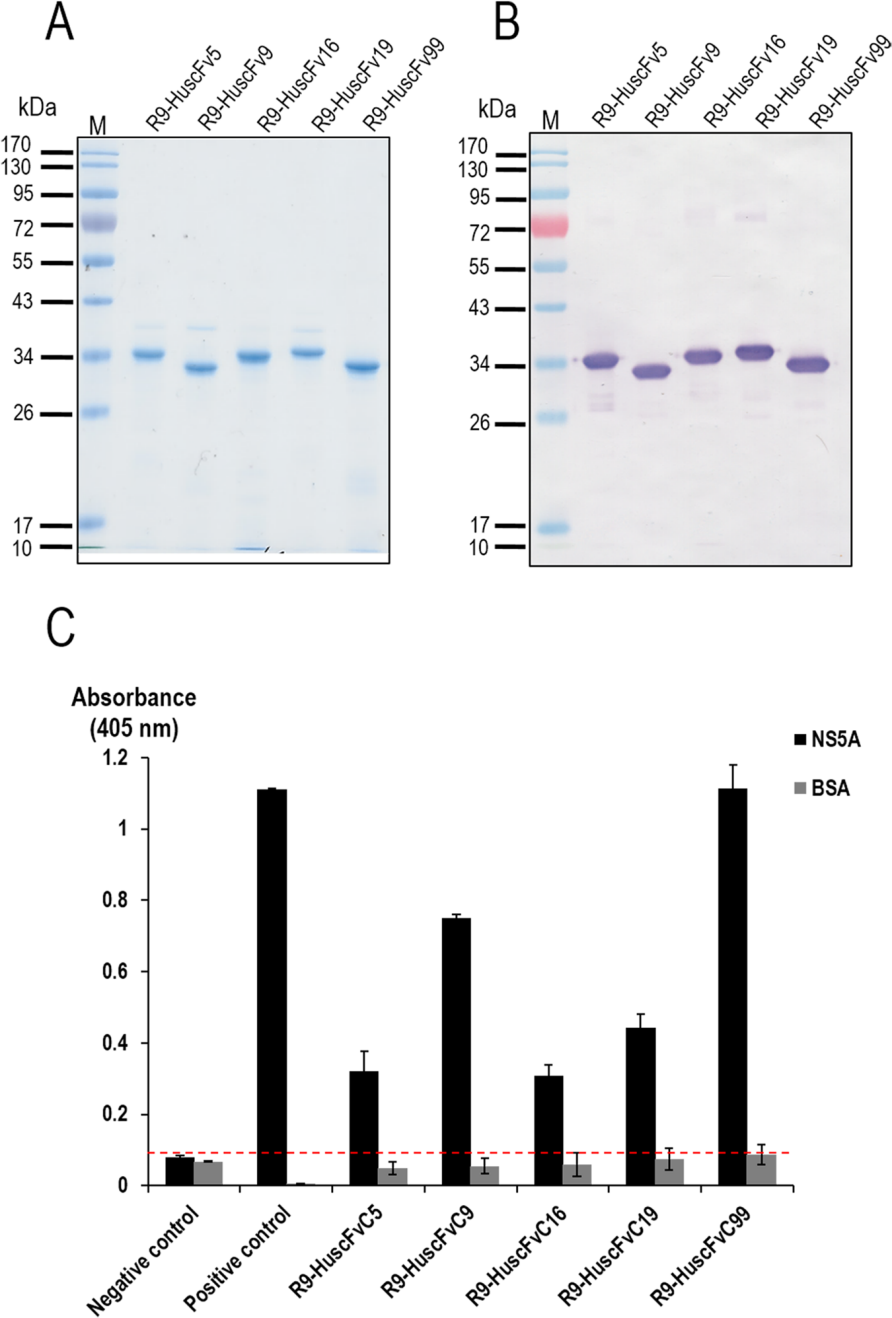
Purified R9-HuscFvs. Panel A depicts SDS-PAGE-separated purified R9-HuscFv5, HuscFv9, HuscFv16, HuscFv19, and HuscFv99 stained with Coomassie Brilliant Blue G-250 (CBB) dye. Panel B illustrates patterns of the purified R9-HuscFvs in the Western blot analysis. Panel C shows indirect ELISA results to demonstrate that the R9-HuscFv fusion proteins still retained the NS5A-binding activity of the original HuscFvs.

**Figure 4:**
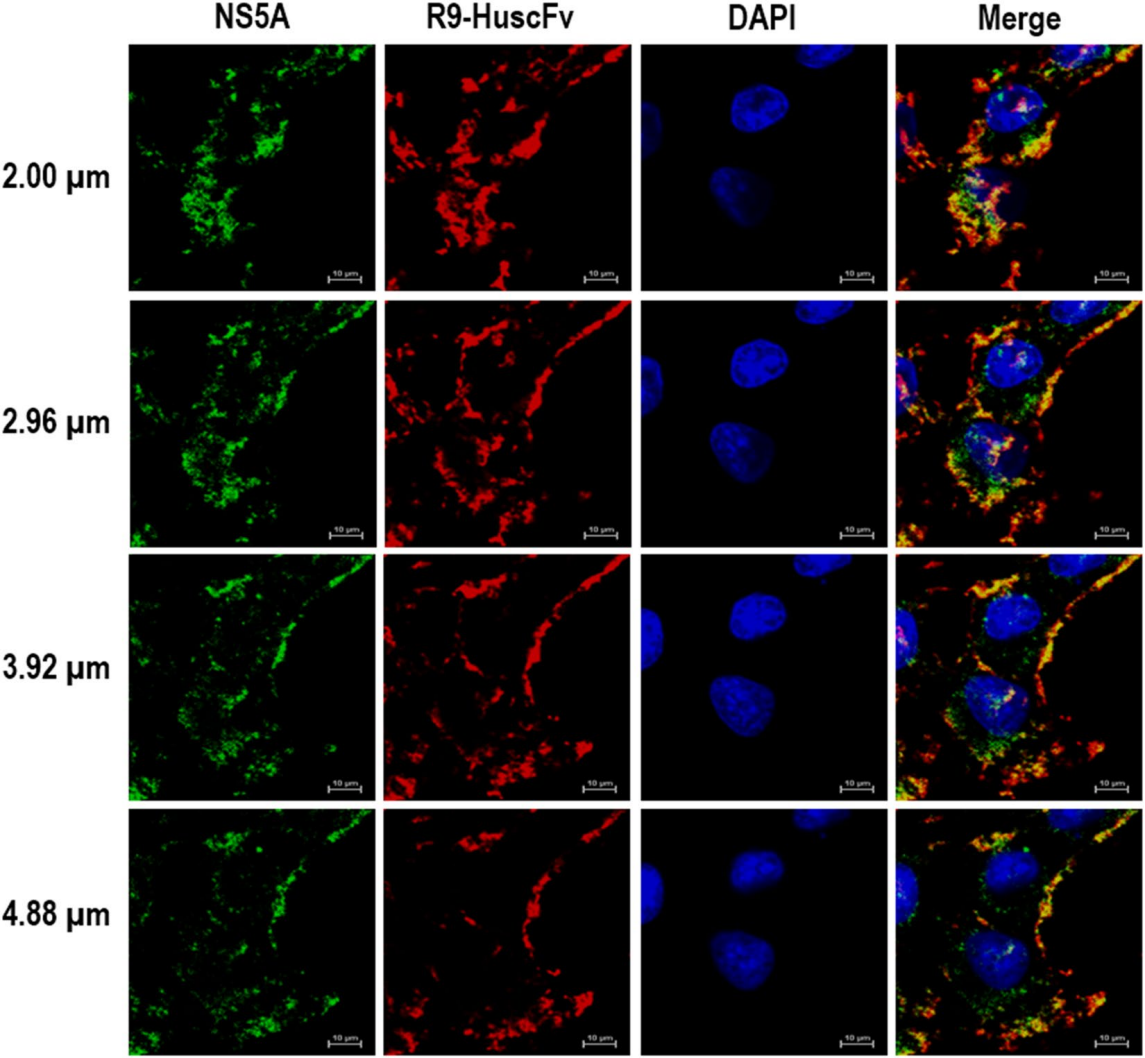
Cellular internalization and intracellular target binding activity of the R9-HuscFvs. Huh7 cells were incubated with R9−HuscFv99 (as representative). After incubation, the cells were fixed, permeated, and stained with rabbit anti-E tag followed by goat anti-rabbit immunoglobulin-AlexaFluor^®^–594 and donkey anti-mouse immunoglobulin-AlexaFluor^®^–488 and counterstained with DAPI for locating nuclei. The preparation was subjected to laser sectional confocal microscopy at 0.96 μm per section, i.e., 2, 2.96, 3.92 and 4.88 μm from top to bottom. Intracellular NS5A appears in green; R9-HuscFv99 in red and nuclei in blue. The farthest right column demonstrates merge of all blocks of the same horizontal row.

In order to interfere with the intracellular NS5A activity of the replicating HCV, the HuscFvs must be able to enter the host cells and interact with the intracellular target. Usually, mammalian plasma membrane is formidable for antibody molecules and conventional antibodies can function only extracellularly^36^. To produce the cell penetrating HuscFvs, gene sequences coding for HuscFvs of the *E. coli* clones 5, 9, 16, 19, and 99 were linked to DNA sequence coding for a cell penetrating peptide (CPP), *i.e*., nonaarginine (R9), by means of the previously described ligase independent cloning (LIC) method^38^. Purified R9-HuscFvs (∼30–36 kDa) of all *E. coli* clones after SDS-PAGE and Coomassie Brilliant Blue G-250 (CBB) staining are shown in Fig. 3. Ability of the R9-HuscFvs to enter the HCV infected human hepatic (Huh7) cells and bound to the intracellular native NS5A target was determined by laser sectional confocal microscopic assay. Figure 4 shows intracellular R9-HuscFv99 (as a representative) in red-fluorescence, intracellularly produced NS5A in green-fluorescence and nuclei in blue. Co-localization of the R9-HuscFv99 and the native NS5A which appeared in yellow/orange after merging at different levels of the cell sections are seen.

### Immunoprecipitation to demonstrate binding of the R9-HuscFvs to native NS5A

**Figure 5:**
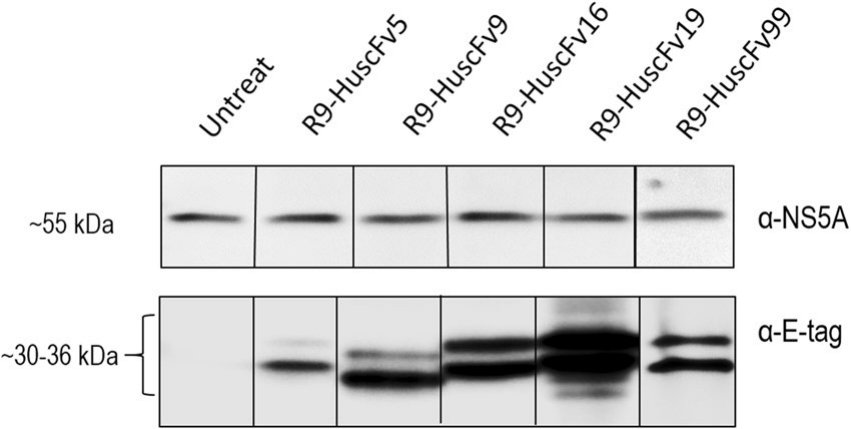
Binding of the R9-HuscFvs to native NS5A as determined by immunoprecipitation test. Lysates of HCV infected Huh7 cells treated with R9-HuscFvs were mixed with protein G resin coated with mouse anti-NS5A antibody and subjected to Western blot analysis. The preparations from lysates of HCV infected cells treated with R9-HuscFv5, R9-HuscFv9, R9-HuscFv16, R9-HuscFv19 and R9-HuscFv99 after SDS-PAGE were found to contain both NS5A (detected by mouse anti-NS5A) and R9-HuscFvs (detected by anti-E tag). The Preparation from HCV infected cells without R9-HuscFv treatment revealed only the NS5A. The results indicate that thr R9-HuscFvs bound to native NS5A.

HCV infected cells that had been exposed to R9-HuscFvs for 3 days were lysed and the NS5A in their lysates were fished-out using Protein-G beads coated with mouse anti-NS5A antibody. The preparations were subjected to Western blot analysis and the results are shown in Fig. 5. Native NS5A in the preparation are seen as protein bands at ∼55 kDa after the SDS-PAGE-separated preparations were probed with mouse anti-NS5A (upper block of Fig. 5). The same preparations also contained the respective R9-HuscFvs (∼30–36 kDa) as detected by rabbit anti-E-tag (lower block of Fig. 5). The preparation derived from lysate of HCV infected cells without R9-HuscFv treatment revealed only the NS5A. The results indicate that the R9-HuscFvs bound to native NS5A.

### Human monoclonal transbodies-mediated HCV replication inhibition

**Figure 6:**
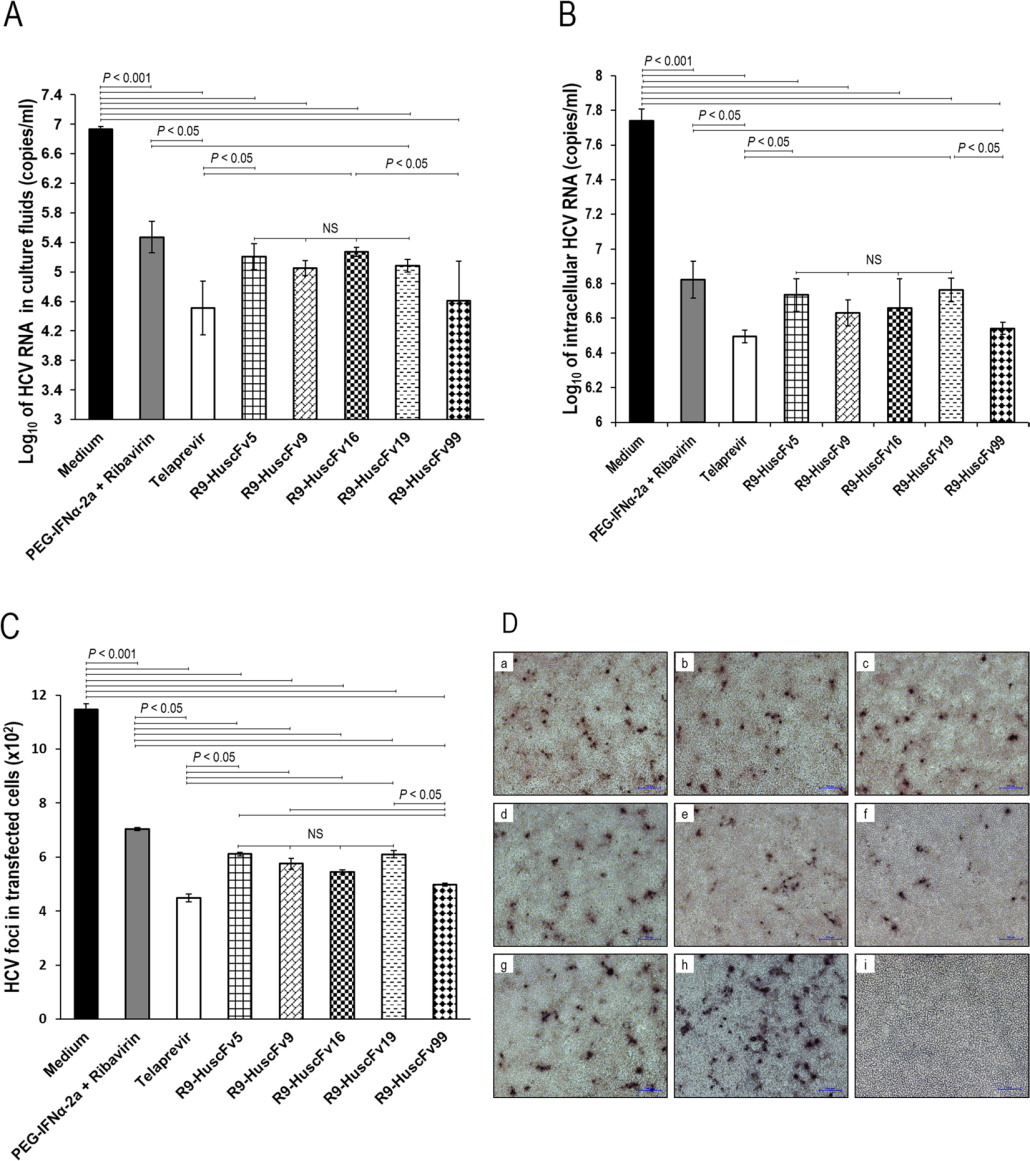
Transbodies-mediated HCV replication inhibition. Panel A shows amounts of HCV 5′-UTR RNA in culture fluids of HCV infected cells after treating with the R9-HuscFv5, R9-HuscFv9, R9-HuscFv16, R9-HuscFv19, and R9-HuscFv99 in comparison with controls. Panel B shows comparative amounts of HCV 5′-UTR RNA in the infected cells after treatments. Panel C shows numbers of HCV foci in the HCV infected cells after being exposed to the R9-HuscFvs in comparison to the controls. Panel D depicts appearance of HCV foci in infected cells after treatments with various HCV inhibitors and controls. a-h, HCV foci in infected Huh7 cells treated with R9-HuscFv5, R9-HuscFv9, R9-HuscFv16, R9-HuscFv19, R9-HuscFv99, telaprevir, pegylated-IFNα-2a + RBV and medium alone, respectively; i, normal Huh7 cells.

Animal model of HCV infection is lacking. However in 2005, the HCV replicon system for HCV replication and production of the infectious viral particles in cell culture (HCVcc) was invented^40–42^. The system has made the investigations on several attributions of the HCV and the HCV infection possible including the viral RNA replication, virus-host interactions, pathogenesis as well as for testing efficacy of innovative HCV inhibitors. HCV replicon was constructed^40^ by cloning the genome of genotype 2a HCV from a Japanese patient with fulminant hepatitis (JFH) into a plasmid. HCV RNA can be generated from the recombinant plasmid, designated pJFH-1, by digesting the plasmid with an endonuclease. The linearized plasmid is transcribed *in vitro* to generate cRNA and the cRNA is introduced into cultured human hepatic cells. The HCV replicates in the cells and infectious HCV particles (HCVcc) can be produced. In this study, ability of NS5A-bound R9-HuscFvs in inhibiting the HCV replication in the HCV infected Huh7 cells was investigated. The HCV infected cells were incubated with R9-HuscFv5, R9-HuscFv9, R9-HuscFv16, R9-HuscFv19, and R9-HuscFv99. Positive inhibitor controls were infected cells incubated with pegylated-IFNα + RBV (SOC) and telaprevir (a chemical inhibitor of HCV protease) and negative inhibitor was infected cells in culture medium alone. After 5 days, amounts of HCV 5′-UTR in the culture supernatants and cells of all treatment groups were determined by quantitative reverse transcription PCR (qRT-PCR)^39,43^. Log_10_ of HCV 5′-UTR (copy numbers/ml) recovered from the culture supernatants and the respective HCV infected cells are shown in Fig. 6A and B, respectively. HCV infected cells in medium alone yielded the most HCV RNA copies in both culture supernatants and inside the cells. The HCV RNAs in culture supernatants and infected cells treated with the R9-HuscFvs and positive inhibitor controls were significantly less than the infected cells in culture medium alone (*p* < 0.001). The R9-HuscFv99 and telaprevir were the most effective inhibitors of the HCV replication; the amounts of HCV RNAs on these two treatment groups were less than the infected cells treated with pegylated-IFNα + RBV and R9-HuscFv19 (*p* < 0.05). The transbodies of the other *E. coli* clones were as effective as the pegylated-IFNα + RBV (SOC) (*p* > 0.05). The R9-HuscFv5, R9-HuscFv9, R9-HuscFv16 and R9-HuscFV19 were equally effective (*p* > 0.05). Overall results indicate that the NS5A-bound R9-HuscFvs caused inhibition of the HCV replication.

Infectious HCV foci formed inside the HCV infected cells of all treatments were determined by using mouse PAb to HCV core protein to probe the viral foci^39^. Results of the HCV foci enumeration (Fig. 6C) conformed to the 5′-UTR data. The infected cells in the medium alone produced many more HCV foci than in the infected cells treated with the R9-HuscFvs and positive inhibitors (*p* < 0.001). The number of HCV foci in the infected cells treated with the R9-HuscFv99 was not different from the telaprevir treated cells (*p* > 0.05) and both were the most effective inhibitors for HCV focal formation. Effectiveness of telaprevir and R9-HuscFv99 was more than the pegylated-IFNα + RBV, R9-HuscFv5, R9-HuscFv9, R9-HuscFv16 and R9-HuscFv19 (*p* < 0.05). Examples of the HCV foci in the infected cells of various treatment groups are shown in Fig. 6D. The finding that the R9-HuscFvs to NS5A could impede the HCV replication led us to investigate further whether or not the transbodies could also rescue the host immunity that had been suppressed by the HCV infection^5^.

### Expression of the innate immune response genes in the HCV infected cells

**Figure 7:**
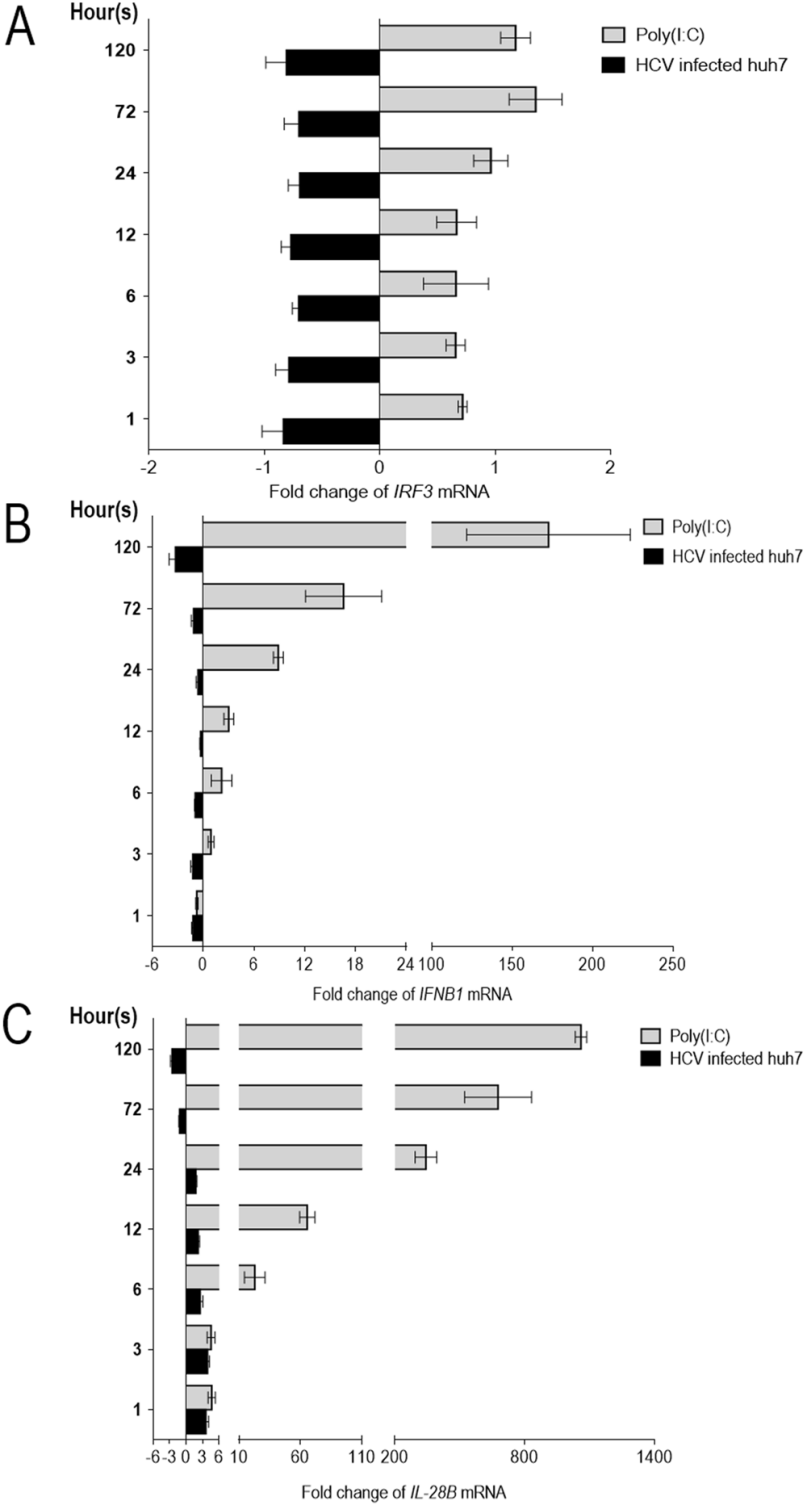
Kinetics of innate gene expression in HCV infected cells. Fold change in expressions of innate immune response genes including *IRF3* (**A**), *IFNB1* (**B**) and *IL-28B* (**C**) in HCV infected Huh7 cells at different times after infection were determined by qRT-PCR using cells stimulated with poly(I:C) as positive control in comparison to the normal cells. Suppression of *IRF3* and *IFNB1* in the HCV infected cells were observed as early as 1 h post infection while the HCV-mediated *IL-28B* suppression was detected at 72 h post infection. The HCV-mediated innate gene down-regulations were pronounced at 120 h post infection. The poly(I:C) started to up-regulate *IRF3, IFNB1* and *IL-28B* at 1, 3 and 1 h post stimulation, respectively; the gene up-regulations by poly(I:C) were observed until the end of the experiments.

Expressions of innate immune response genes including *IRF3* (coding for type-1 interferon transcription factor)*, IFNB1* (type I interferon gene) and *IL-28B* (type III interferon gene) in the HCV infected hepatic cells and the hepatic cells stimulated with poly(I:C) at different times (1, 3, 6, 12, 24, 72 and 120 h) were determined by qRT-PCR in comparison to the uninfected cells. The RNA (300 ng) extracted from the infected/poly(I:C) treated cells was used as template for the qRT-PCR. GAPDH RNA was used for normalization. The results showed that cells stimulated with poly(I:C) started to have up-regulation of *IRF3, IFNB1* and *IL-28B* at 1, 3 and 1 h, respectively, and the magnitudes of the responses increased at the later time points (Fig. 7A–C, respectively). On contrary, cells infected with HCV had down-regulation of the *IRF3* and *IFNB1* since the first hour of infection (Fig. 7A and B) while HCV-mediated suppression of the *IL-28B* was observed at 72 and 120 h post-infection (Fig. 7C). Suppression of the innate gene responses by the HCV was pronounced at day 5 post-infection. The data indicate that HCV could inhibit the host innate immune response gene expressions, most probably by using the early produced NS3/4A protease that has been known to cleave TRIF and Cardif/MAVS, binds to TBK1, and inhibits activation of innate interferon signaling pathways^44–46^.

### Response of HCV infected cells to treatment with the R9-HuscFvs

**Figure 8:**
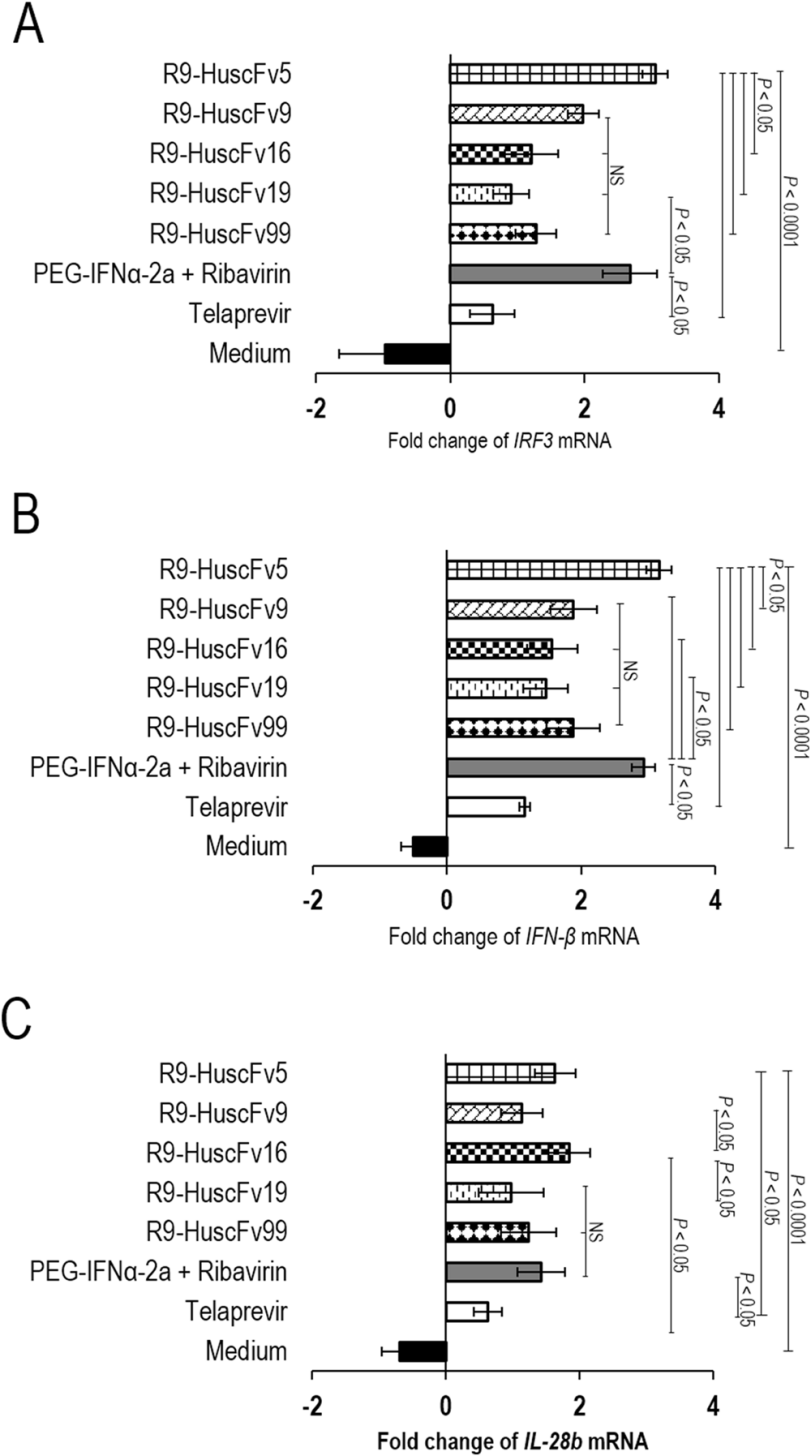
Fold change of mRNA expressions of the innate immune response genes in HCV infected cells after treating with the NS5A-bound-transbodies and controls (pegylated-IFNα-2a + RBV, telaprevir and medium alone) in comparison to normal (non-infected) cells. Panels A–C show fold change of expressions of *IRF3, IFN-B* and *IL-28*, respectively, after exposure to the transbodies and controls. The NS5A-bound-R9-HuscFvs and positive inhibitor controls restored the expressions of the innate genes that had been suppressed by the replicating HCV. All studied genes in the HCV infected cells cultured in medium alone were down-regulated by the infection.

RNAs were isolated from the HCV infected cells after the cells had been exposed for 5 days to the R9-HuscFvs and controls. Expressions of the studied innate immune response genes in the transbody-treated HCV infected cells and controls in relation to normal cells are shown in Fig. 8A–C, respectively. The R9-HuscFvs and the positive HCV replication inhibition controls (pegylated-IFNα + RBV and telaprevir) effectively restored the host gene expressions while apparent immune suppression was retained in the HCV infected cells kept in the medium alone (*p* < 0.0001). The data indicate that the R9-HuscFVs not only inhibited the viral replication, but also restored the host innate immune response. The transbodies should be relatively safe if used in treatment of HCV infection in humans (no anti-isotype/anti-R9 response in the treated human subjects). Usually, an antibody molecule uses several residues in their multiple CDR loops to cooperate in target binding. Such multiple site-binding should render difficulty for the HCV to mutate and create the transbody-escape, functional NS5A mutant. This is in contrast to the HCV small chemical inhibitors, such as telaprevir (HCV protease inhibitor) and sofosbuvir (NS5B polymerase inhibitor) which drug escape HCV mutants emerged rapidly by a single amino acid mutation of the target proteins^47–50^.

### Computerized simulation for determining regions and residues of the NS5A that were bound by the HuscFvs

**Figure 9:**
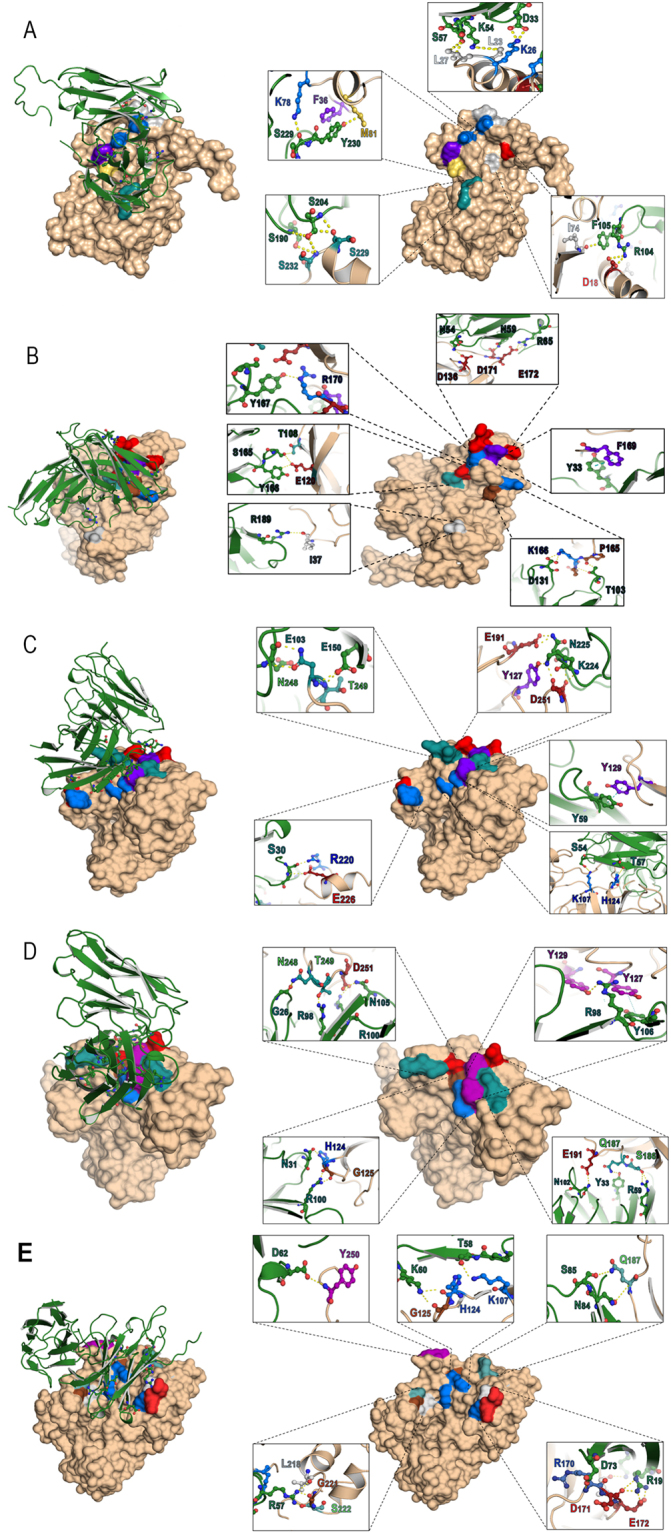
Computerized interaction of modeled-NS5A and HuscFvs and residues that were predicted to form contact interface between them. Left side of Panels A-E shows interactions of NS5A (beige) and respective HuscFvs (green). Right side of Panels A-E shows contact residues between NS5A and HuscFv5, HuscFv9, HuscFv16, HuscFv19 and HuscFv99, respectively. The NS5A amino acids are colored according to CINEMA color scheme: polar negative D and E are red; polar positive H, K, and R are blue; polar neutral S, T, N, and Q are green; non-polar aromatic F and Y are purple/magenta; non-polar aliphatic A, V, L, I, and M are white (grey in this study as the background is white); and P and G are brown. The π-π interaction is shown as dotted green line; H bond/salt-bridge is shown as dotted yellow line.

Computerized simulation was performed to determine the presumptive NS5A regions and residues that formed contact interface with the effective HuscFvs in order to gain some insight into the mechanisms of the HuscFv-mediated HCV replication inhibition. From the homology modeling and intermolecular docking, the complexes formed between the modeled HCV NS5A and HuscFv5, HuscFv9, HuscFv16, HuscFv19, and HuscFv99 that revealed the largest interactive clusters with the lowest local energy scores were selected. According to the docking outputs, all HuscFvs were predicted to form contact interface mainly with domain-I of the NS5A (Table 1) and Fig. 9. It is known that N-terminal residues 5–25 of NS5A form amphipathic helix (AH) which anchors the protein to the web membrane for the RC formation^31,32^. This region also coordinates a zinc atom to the NS5A molecule^33^. Mutation disruption of either one of these two N-terminal activities inhibits HCV replication (hence lethal mutations)^33^. The HuscFv5 was predicted to interact with a conformational epitope formed by two NS5A N-terminal regions [residues D18 and L23 of AH and K26, L27, F36, I74, K78 and M81 of domain-I) and S229 and S232 of LCS-I] that are spatially juxtaposed upon the protein folding (Fig. 9A). Binding of the HuscFv5 to the AH and domain-I should interfere with both the NS5A localization at the web and the zinc ion coordination and should lead to impairment of HCV replication. Besides, the NS5A molecule is known to contain several proline-rich motifs, either class-I ( + XϕPXϕP) or class-II (ϕPXϕPX)^48^. The class-II motifs interact with cellular Src-homology 3 (SH3) adapter proteins and modulate several cellular activities through the respective SH3 downstream signaling pathways including mitogenicity, apoptosis, stress response, protein subcellular localization and cytoskletal organization^51–54^. Although the important role of the highly conserved class-I proline rich-motif at residues 26–32 of the NS5A has not yet been elucidated, it is plausible that presumptive binding of the R9-HuscFv5 to K26 and L27 of this motif should have some impact on the unknown activity of the NS5A which should be incompatible with the HCV infectious cycle. The S229 and S232 of the LCS-I are the main hyperphosphorylation sites of the NS5A. Hyperphosphorylated NS5A (p58) has a suggestive role in HCV replication regulation^54^. Binding of the R9-HuscFv5 to these sites may perturb the p58 activity. Moreover, N-terminal residues 1–148 of the NS5A confers innate immune evasion for the HCV by interacting with 2′,5′-oligoadenylate synthetase (2′,5′-OAS), an important anti-viral protein^55^. All VH-CDRs and VL-CDR3 of the HuscFv5 were predicted to cooperatively formed contact interface with several NS5A N-terminal residues which should interfere with the NS5A-2,5′-OAS interaction and consequently restored the host innate immunity. This speculation is fully supported by the finding that expressions of *IRF3*, *IFNB1* as well as *IL-28B* were regained and even enhanced in the R9-HuscFv5-treated HCV infected cells, compared to the untreated infected Huh7 and the uninfected (normal) cells.

**Table 1:** Presumptive residues and regions of HCV NS5A bound by the HuscFvs as determined by computerized simulation.

HCV NS5A protein		HuscFv5		
Residue	Domain	Residue(s)	Domain	Interactive bond
D18	AH of domain I	R104	VH-CDR3	Salt bridge
L23	AH of domain I	K54	VH-CDR2	H-bond
K26	Domain I	D33	VH-CDR1	Salt bridge
L27	Domain I	S57	VH-CDR2	H-bond
F36	Domain I	Y230	VL-CDR3	π-π interaction
I74	Domain I	F105	VH-CDR3	H-bond
K78	Domain I	S229	VL-CDR3	H-bond
M81	Domain I	Y230	VL-CDR3	H-bond
S229	LCS-I	S204	VL-FR3	H-bond
S232	LCS-I	S204/S190	VL-FR3	H-bond
**HCV NS5A protein**		**HuscFv9**		
**Residues**	**Domain**	**Residue(s)**	**Domain**	**Interactive bonds**
I37	Domain I	R189	VL-FR3	H-bond
T108	Domain I	S165	VL-CDR1	H-bond
E120	Domain I	Y167	VL-CDR1	H-bond
D136	Domain I	N54	VH-CDR2	Salt bridge
P165	Domain I	T102	VH-CDR3	H-bond
K166	Domain I	D31	VH-CDR1	Salt bridge
F169	Domain I	Y33	VH-CDR2	π-π interaction
R170	Domain I	Y167	VL-CDR1	H-bond
D171	Domain I	N59	VH-CDR2	H-bond
E172	Domain I	R65	VH-FR3	Salt bridge
**HCV NS5A protein**		**HuscFv16**		
**Residues**	**Domain**	**Residue(s)**	**Domain**	**Interactive bond**
K107	Domain I	S54	VH-CDR2	H-bond
H124	Domain I	T57	VH-CDR2	H-bond
Y127	Domain I	K224	VL-CDR3	Salt bridge
Y129	Domain I	Y59	VH-CDR2	π-π interaction
E191	Domain I	N225	VL-CDR3	H-bond
R220	Domain I	S30	VH-CDR1	H-bond
E226	Domain I	S30	VH-CDR1	H-bond
D251	Domain II	K224	VL-CDR3	Salt bridge
**HCV NS5A protein**		**HuscFv19**		
**Residues**	**Domain**	**Residue(s)**	**Domain**	**Interactive bond**
H124	Domain I	N31	VH-CDR1	H-bond
G125	Domain I	R100	VH-CDR3	H-bond
Y127	Domain I	Y106	VH-CDR3	H-bond
Y129	Domain I	R98	VH-CDR3	H-bond
S186	Domain I	R59	VH-FR3	H-bond
E191	Domain I	N102	VH-CDR3	H-bond
N248	LCS-I	G26	VH-CDR1	H-bond
T249	LCS-I	R98	VH-CDR3	H-bond
D251	Domain II	R100/N105	VH-CDR3	Salt bridge
**HCV NS5A protein**		**HuscFv99**		
**Residues**	**Domain**	**Residue(s)**	**Domain**	**Interactive bond**
K107	Domain I	T58	VH-CDR2	H-bond
H124	Domain I	K60	VH-FR3	H-bond
G125	Domain I	K60	VH-FR3	H bond
R170	Domain I	D73	VH-FR3	Salt bridge
D171	Domain I	R19	VH-FR1	Salt bridge
E172	Domain I	R19	VH-FR1	Salt bridge
Q187	Domain I	N84/S85	VH-FR3	H-bond
L218/	LCS-I	R57	VH-CDR2	H-bond
G221	LCS-I	R57	VH-CDR2	H-bond
S222	LCS-I	R57	VH-CDR2	H-bond
Y250	Domain II	D62	VH-FR3	H-bond

LCS-1, low complexity sequence between domains I and II of NS5A.

NS5A domain-I binds RNA for HCV replication^33^. Residues 105–162 of this domain interact with HCV NS5B polymerase and regulate the viral replication^56,57^. Residues 163–167 of NS5A bound to NS4B for RC formation while residues 1–83 bind to La (LARP3) which is a multifunctional cellular phosphoprotein that involves in several cellular metabolisms and viral RNA activities, such as RNA binding and chaperoning, dsRNA unwinding (helicase activity), mRNA stabilization, RNA polymerase transcripts and cellular microRNA processing, and binding to IRES to mediate HCV translation^54,58–62^. Residues 1–181 of domain-I bind to cellular heat shock response protein (HSP27) and residues 1–110 bound to p85 of cellular phosphoinositide 3-kinase causing up-regulation of the PI3K/Akt cellular survival pathway, possibly for HCV persistence and pathogenesis^63–65^. Moreover, NS5A residues 123–131 and 155–172 contain Bcl-2 homology (BH) domains 3 and 1, respectively, which protect cells against apoptosis and thus in favor of the HCV replication and pathogenesis^66^. The HuscFv9, HuscFv16, HuscFv19 and HuscFv99 were predicted to form contact interface with T108, E120, D136, P165, K166, F169, R170, D171 and E172 (Fig. 9B); K107, H124, Y127, Y129, and E191 of domain-I and R220 and E226 of LCS-1 (Fig. 9C); H124, G125, Y127, Y129, S186, Q187, and E191 of domain-I, N248 and T249 of LCS-1, and D251 of domain-II (Fig. 9D); and K107, T108, H124, G125, Y129, R170, D171, E172, and Q187 of domain-I, L218, G221, and S222 of LCS-1, and Y250 of domain-II (Fig. 9E), respectively. The interactions should not merely interfere with the NS5A binding to the 2′5′-OAS and thus rescued the host response as mentioned earlier, but should also perturb the viral replication and other NS5A bioactivities of the NS5A N-terminal regions.

### Epitope mapping of the R9-HuscFvs to NS5A

**Figure 10:**
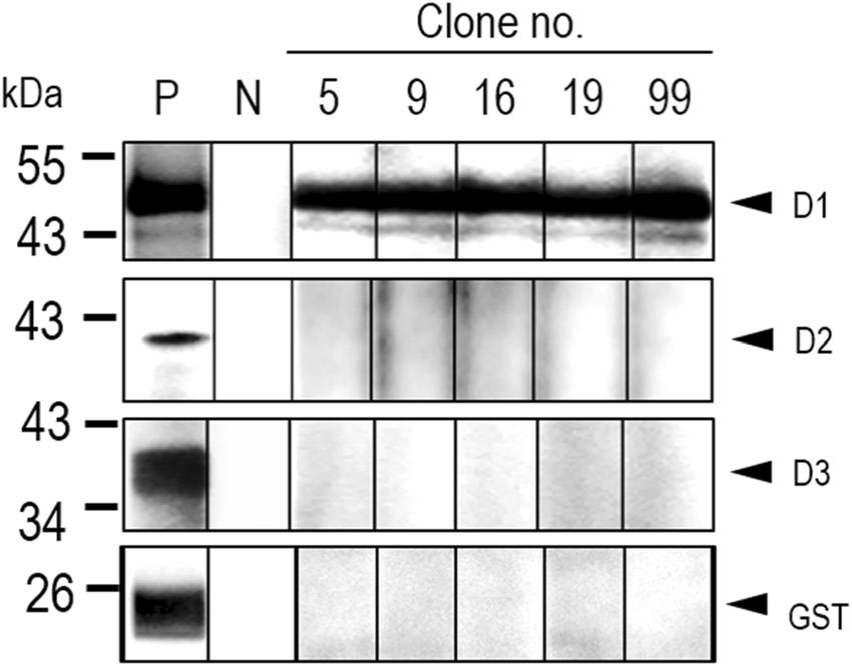
Western blot analysis to determine NS5A domain bound by R9-HuscFvs. SDS-PAGE-separated GST-tagged-recombinant D1, D2, and D3 of NS5A and GST were probed with E-tagged R9-HuscFvs, followed by rabbit anti-E-Tag, goat anti-rabbit isotype-HRP conjugate and substrate, respectively. N, negative binding controls (antigen-blotted strips were incubated with buffer instead of the respective R9-HuscFv). P, positive controls (antigen-blotted strips were probed with goat-anti-GST). All of the R9-HuscFvs bound to the NS5A D1 and did not react to the D2 and D3 or GST control.

In order to verify the computerized intermolecular docking results which indicated that the R9-HuscFvs bound mainly to the NS5A domain-I, Western blotting were performed using the recombinant GST-tagged D1, D2, and D3 as the antigens. The results shown in Fig. 10 verified the computerized results that all R9-HuscFvs gave positive binding to the NS5A D1 and did not react to the D2 and D3 proteins or the GST tag.

### Concluding remarks

Human monoclonal single chain-transbodies (nonaarginine-linked HuscFvs) to NS5A which is an enigmatic and multifunctional protein in the HCV replication cycle and pathogenesis were produced. The cell penetrating small antibodies inhibited the HCV replication and readily rescued the host innate immune response genes from the viral suppression. Computerized simulation and epitope mapping by immunoassay demonstrated that the effective transbodies interacted mainly with the NS5A domain-I pivotal for HCV replication complex formation, RNA binding, and interaction with several host proteins for viral immune evasion and cellular physiology regulation. Although their molecular mechanisms leading to the observed outcomes await detail laboratory investigations, the cell penetrable HuscFvs have high potential for developing and testing further as a safe, direct acting (possibly interferon free-) anti-HCV agent. To our knowledge, this is the first report on human monoclonal transbodies that interfere with the HCV NS5A activities leading to effective HCV replication inhibition and host immunity restoration.

## Methods

### Production of recombinant NS5A and D1, D2 and D3

Coding sequence of NS5A was amplified from pJFH-1 (genotype 2a) using PCR primers designed from a database sequence (accession no. AB47639). The forward primer was 5′-CCCGGCCTCCCCTTCATCTC-3′ and the reverse primer was 5′-ATCCCGACCCATTTGCTGTCCAC-3′. The PCR reaction mixture (25 μl) contained 2.5 μl 10 × polymerase buffer; 1.5 μl 25 mM MgCl_2_; 1 μl 10 mM dNTP mixture; 0.1 μl DNA polymerase; 1 μl DNA template, and ultrapure distilled water. The thermal cycles were initial denaturation at 95 °C, 10 min; 30 cycles of denaturation at 95 °C for 1 min, annealing at 62 °C for 1 min and extension at 72 °C for 1 min; and the last extension at 72 °C, 10 min. The DNA amplicon was cloned into pTZ52R/T, subcloned to pET32 protein expression vector, and electroporated into BL21 (DE3) *E. coli*. Transformed *E. coli* colonies grown on selective Luria-Bertani (LB) agar plates containing 100 μg/ml ampicillin (LB-A) were screened by direct colony PCR for plasmid carrying the NS5A gene insert. Appropriately transformed bacterial colony was grown in LB-A broth containing 0.5 mM IPTG for about 5 h. The bacterial pellet was homogenized by sonication and the 6× His-tagged-NS5A was purified from the homogenate by using HisTrap FF column (GE Healthcare). The recombinant protein was verified by mass spectrometry.

PCR primers for amplification of DNAs coding for NS5A D1 (residues 32–213), D2 (residues 249–337) and D3 (residues 351–465) are listed in Supplementary Table 1. The pJFH-1 was used also as the template. The PCR was carried out using high fidelity Q5 DNA polymerase (New England Biolab, USA) according to the instruction manual. The gene amplicons were ligated individually to pTZ57R/T cloning vector and subcloned to pGEX4T-1. The recombinant vectors were introduced separately into BL21 *E. coli*. Bacterial transformants carrying the correct recombinant plasmids (verified by DNA sequencing) were grown in 0.1 mM IPTG supplemented-LB-A broth for 3 h. Bacterial pellets were homogenized by sonication, centrifuged, and both soluble and insoluble fractions were subjected to Western blot analysis using rabbit anti-GST antibody for probing the GST-tagged-recombinant proteins. The D1 protein was found in the bacterial insoluble fraction while the D2 and D3 were soluble. Thus, the D2 and D3 were purified from the respective bacterial lysates by using GSTrap column (GE Healthcare) according to the instruction manual. The D1 was purified from the bacterial inclusion body (IB) as described previously^39^. Briefly, a bacterial colony carrying recombinant *d1-*plasmid was grown under 0.1 mM IPTG induction in 250 ml of 2× yeast-tryptone (2× YT) broth containing 100 μg/ml ampicillin at 30 °C with shaking (250 rpm) for 5 h. One gram of the bacterial pellet collected after centrifugation was suspended in 5 ml of BugBuster^®^ Protein Extraction Reagent (Novagen, EMD Millipore, MA, USA) and 20 μl of Lysonase^TM^ Bioprocessing Reagent (Novagen) were added. The preparation was kept shaking (250 rpm) at 25 °C for 20 min and centrifuged at 8000 × *g* at 4 °C for 30 min. The IB containing recombinant D1 protein was washed twice with Wash-100 buffer, twice with Wash-114 buffer, suspended in a Wash solvent and kept shaking at 250 rpm for 20 min. The pellet was suspended in sterile distilled water, washed, and adjusted to 50 μg/ml. Purity of the preparation was checked by SDS-PAGE and protein staining. Purified IB was subjected to protein refolding as described previously^39^.

### Preparation of polyclonal antibody (PAb) to HCV core protein and NS5A

Animal experiments were carried out following Guideline of the National Research Council of Thailand and received approval from the Animal Care and Use Committee of Siriraj Hospital, Mahidol University, Bangkok (SI-ACUP 016/2557). Two groups of three ICR mice were injected individually and intraperitoneally with either 10 μg of recombinant core protein or NS5A mixed with alum adjuvant (1:3 v/v). Three booster doses were given to the primed animals at 14 day intervals using the same dose of the respective immunogens and the same route. One week after last booster, mice were bled and immune sera of individual groups were collected and pooled separately. The effective concentration-50 (EC_50_) of the anti-core and anti-NS5A immune serum pools were determined by indirect ELISA using 1 μg of the homologous antigens to coat individual wells of the ELISA plate. The immune serum pools were kept in small aliquots at −20 °C until use.

### Production of human monoclonal scFvs (HuscFvs) that bound to NS5A

The rNS5A was used as antigen in phage biopanning for selecting phages that bound to the protein from a human scFv-phage display library^67^. The panning was performed as described previously^43^. The NS5A-bound phage clones were used to transfect the F+ non-suppressor HB2151 *E. coli* and the phage-transformed bacteria were screened for HuscFv coding sequences (*huscfvs*) by PCR using the pCANTAB-5E phagemid specific primers^67^. The *huscfv*-positive bacterial colonies were grown under IPTG-induction condition and their lysates were tested for the presence of E-tagged-HuscFvs by Western blotting using rabbit anti-E-tag (Abcam, UK) as the HuscFv tracing reagent. HuscFvs in homogenates of the positive *E. coli* clones were standardized spectrometrically based on the band intensity on the Western blot membrane before testing for binding to the panning antigen by indirect ELISA using one μg of rNS5A to coat each ELISA well. BSA and lysate of original HB2151 *E. coli* clone (HB) were included in the ELISA as control antigen and background binding control, respectively. Nucleotide sequences of *huscfvs* coding for NS5A-bound HuscFvs were determined and deduced. Complementarity determining regions (CDRs) and canonical framework regions (FRs) of individual sequences were worked out using the International Immunogenetics Information system (www.imgt.org).

### Preparation of cell penetrable HuscFvs (transbodies)

Gene sequences coding for NS5A-bound HuscFvs were linked to a sequence coding for nonaarginine (R9). The PCR amplified *huscfvs* were cut with *Not*I and *Sfi*I restriction enzymes and ligated to pLATE52 vector via the similarly cut sites. The recombinant plasmids were introduced into JM109 *E. coli* (K12) for improving yield and quality of the DNAs before transfecting into Rosetta^TM^ (DE3) *E. coli*. For large scale production of R9-HuscFvs, individual Rosetta *E. coli* clones carrying *R9-huscfv*-plasmids were grown under 1 mM IPTG induction in 250 ml of 2× YT broth containing 100 μg/ml ampicillin and 34 μg/ml chloramphenicol at 30 °C with shaking (250 rpm) for 6 h. The bacterial IB containing the R9-HuscFvs were purified from the respective bacterial IBs as described above for preparing the recombinant NS5A D1. The R9-HuscFvs were retested for binding to the NS5A protein by Western blot analysis and indirect ELISA.

### *In vitro* transcription and preparation of HCV infected cells

HCV cRNA was prepared from *in vitro* transcription of the pJFH-1 as described previously^68^. Briefly, the pJFH-1 was digested by *Xba*I enzyme. T7 transcription kit (MEGAscript®, Ambion, Life Technologies, CA, USA) was used for the *in vitro* transcription of the linearized-plasmid to cRNA. The JFH-1 cRNA was introduced into the Huh7 cells by electroporation method and the cells were added immediately with DMEM supplemented with fetal bovine serum (Hyclone, USA), antibiotics, and L-glutamine (complete DMEM). HCV infected cells were cultured in complete DMEM in T160 cell culture flask at 37 °C in 5% CO_2_ atmosphere for 5 days. The culture supernatant containing HCVcc was kept in small aliquots at −80 °C until use.

### Confocal microscopy for determining intracellular localization of the R9-HuscFvs

For testing intracellular localization and target binding ability of the R9-HuscFvs, HCV infected cells maintained in complete DMEM on cover slips (1 × 10^5^ cells/slip) in wells of a 24-well tissue culture plate were added with 20 μg of R9-HuscFvs from individual *E. coli* clones in triplicate and incubated for 3 h. After discarding the culture fluids and rinsing the cells with sterile PBS, the cells were fixed and permeated with ice-cold methanol, then washed with sterile PBS, blocked with 1% BSA in PBS, and washed again. Rabbit anti-E tag (Abcam) (300 μl of 1:3,000 dilution) and mouse anti-NS5A immune serum pool (300 μl of 1:300 dilution) were added to the permeated cells and incubated for 1 h. Goat-anti-rabbit immunoglobulin-AlexaFlour^®^-594 (Life Technlogies, USA) (300 μl of 1:300 dilution) and donkey anti-mouse immunoglobulin-AlexaFlour^®^-488 (Abcam) (300 μl of 1:300 dilution) were used for locating the R9-HuscFvs and NS5A, respectively. DAPI was used for nuclei staining. The stained cells were subjected to laser sectional confocal microscopy.

### Co-immunoprecipitation assay

Binding of the R9-HuscFvs to native NS5A was demonstrated by co-immunoprecipitation assay. Huh7 cells in complete DMEM were seeded into 6-well plate (1.2 × 10^6^ cells/well) and incubated at 37 °C in 5% CO_2_ incubator overnight. After washing, the cells were added with HCVcc in complete DMEM at MOI 5 and incubated further for 3 more days. R9-HuscFv (50 μg) was added to each well and incubated for 3 h. The culture fluids were discarded and the cells were washed three times with PBS. Five hundred μl of lysis buffer (50 mM Tris-HCl, pH 8.0; 150 mM NaCl and 1% Triton X-100) were added to individual wells. The content in each well was transferred to a microcentrifuge tube and centrifuged (10000 × *g*, 4 °C, 5 min). The supernatant was added with 5 μl of mouse anti-NS5A (diluted 1:100 in lysis buffer) and kept at 25 °C for 1 h. Protein-G beads (GE Healthcare) (50 μl) were added to the preparation and kept rotating at 4 °C overnight. The beads were set by centrifugation, washed with lysis buffer three times and added with 100 μl of sample buffer (0.5 M Tris-HCl, pH 8.8; 1% SDS, 5% glycerol and 1% Bromophenol Blue), boiled for 10 min and subjected to SDS-PAGE and Western blot analysis by probing with rabbit anti-E-tag (to detect the R9-HuscFvs) and mouse anti-NS5A.

### Kinetics of innate immune gene expression of the HCV infected cells

Huh7 cells in complete DMEM medium were seeded into wells of a 12-well tissue culture plate (2 × 10^5^ cells/well) and incubated at 37 °C in CO_2_ atmosphere overnight. The cells in each well were infected with HCVcc at MOI 1.0 and incubated. Infected cells from triplicate wells at indicated time points (1, 3, 6, 12, 24, 72 and 120 h post infection) were washed and RNA was extracted by adding 500 μl TriZol reagent. Huh7 cells added with complete DMEM containing 10 μg/ml poly(I:C) were used as positive control. Expressions of host innate immune response genes (*IRF3*, *INFB1* and *IL-28B*) in the HCV infected and poly(I:C) stimulated cells were determined by qRT-PCR using mRNA levels of the naïve Huh7 cells as baseline.

### Inhibition of HCV replication by NS5A-bound transbodies

HCV infected cells in plain DMEM were seeded into wells of a 12-well tissue culture plate (2 × 10^5^ cells/well) and incubated at 37 °C in CO_2_ atmosphere for 6 h. After discarding the culture fluids, complete DMEM containing 30 μg of individual R9-HuscFvs were added appropriately to the cells. Pegylated-IFNα-2α (100 IU) + RBV (50 nM) and 0.175 μM telaprevir (VX-950; Selleckem, Houston, TX, USA) served as positive HCV replication inhibition controls. Negative inhibition control was HCV infected cells in medium alone. The treated infected cells were incubated for 5 days. Total RNAs were isolated from culture fluids and cells of individual wells by using TRIzol^®^ reagent (Ambion). The RNAs were used for HCV 5′-UTR enumeration by quantitative reverse transcription-PCR (qRT-PCR). Alternatively, numbers of HCV foci formed in the HCV infected cells of all treatments were determined by a foci assay.

### Quantitative reverse transcription-PCR (qRT-PCR)

Quantification of HCV 5′-UTR was performed by qRT-PCR as described previously^ 64^. Briefly, PCR reaction mixture (12.5 μl) which contained 6.25 μl 2 × Brilliant II SYBR Green QRT-PCR master mix, 0.5 μl each of the 5′-UTR primers, 0.5 μl RT/RNS block enzyme, 4.75 μl RNA template or 200–900 ng of standard pJFH-1 RNA and sterile DEPC treated-distilled water was prepared. The amplification was carried out using Mx3000P QPCR System (Agilent Technologies). A dissociation curve was analyzed as follows: thermal profile of 95 °C for 1 min, ramped down to 55 °C for 45 s at a speed of 0.5 °C/s, and ramped up to 95 °C. Then Log_10_ RNA copies/ml of each sample was extrapolated from the standard curve which was built from cycle threshold (C*t*) of ten-fold serially diluted pJFH-1 (full-length cDNA HCV genotype 2a) which were cal. 2.77 × 10^7^ to 0.02 DNA copies.

### HCV foci assay

Numbers of HCV foci of the HCV infected cells treated with NS5A-bound R9-HuscFvs and controls were determined^39^. Treated cells in each well were incubated with 100 μl of 1:200 mouse PAb to HCV core protein [effective concentration-50 (EC_50_) of the anti-HCV core immune serum pool was 1:5,000]. After washing, alkaline phosphatase (AP) conjugated-goat anti-mouse isotype (500 μl of 1:3,000 dilution) and 500 μl of AP chromogenic substrate (KPL) were used to reveal the foci. HCV infected cells in medium alone and uninfected (normal) cells were included in the experiments. Enumeration of the foci was performed using an inverted fluorescence microscope (NIS-Element D version 4.10.0.8310 W/camera, Ti-S Intensilight Ril NIS-D, Nikon, Japan) at 40× magnification.

### Response of HCV infected cells to treatment with NS5A-bound R9-HuscFvs

RNAs isolated from the HCV infected cells after treating with NS5A-bound R9-HuscFvs and controls (300 ng each) were used as templates for quantification of innate immune response genes including *IRF3, IFNβ1*, and *IL-28B* by qRT-PCR. GAPDH RNA was used for normalization. The qRT-PCR was performed as for quantification of the HCV 5′-UTR described above using primers specific to the innate genes^39^. C*t* of individual genes were compared with the house keeping gene (Δ*Ct*) and subtracted by background Δ*Ct* of normal cells. Data (ΔΔ*Ct*) are expressed as fold change of individual genes in the HCV infected cells treated with the R9-HuscFvs and controls in comparison to the normal cells.

### Computerized simulation for determining presumptive NS5A regions and residues bound by the HuscFvs

Three dimensional model of the HCV NS5A molecule was acquired from template-based protein structure modeling using RaptorX webserver^69^. The sequence of the pJFH-1 NS5A showed the highest homology with PDB 3FQM. Validation of the acquired model was performed according to the criteria of VADAR version 1.8^70^. The HuscFv sequences were submitted to I-TASSER service for modeling and the models were subsequently refined by ModRefiner and Fragment Guided Molecular Dynamics (FG-MD) simulation for making them become closer to their native state^71–73^. The modeled NS5A and the HuscFvs were subjected to ClusPro2.0 server for determining their contact interface^74^. The intermolecular docking which showed the largest cluster of interactive residues with the lowest local energy was selected. Pymol software (The PyMOL Molecular Graphics System, Version 1.3r1 edu, Schrodinger, LLC, NY, USA) was used for building the molecular interactive protein structure models.

### Western blot analysis for determining the NS5A domain bound by the R9-HuscFvs

Recombinant D1, D2, and D3 proteins with GST-tag were subjected to SDS-PAGE and the separated components were electroblotted onto nitrocellulose membranes (NC). GST was included as antigen control. After blocking the NC empty sites with 5% skim milk, the membranes were air-dried and cut vertically into strips. Individual strips were incubated appropriately with the E-tagged-R9-HuscFvs. After incubating and washing, all NC strips were probed with rabbit monoclonal anti-E tag antibody (Abcam). Horseradish peroxidase (HRP)-conjugated-goat anti-rabbit isotype (Southern Biotech, Bermingham, USA) and HRP substrate (Luminata^TM^ Crescendo Western HRP Substrate; Merck Millipore, MA, USA) were used for revealing the antigen-antibody reactive bands on the membrane. Negative controls (N) were respective antigen-blotted strips probed with buffer while positive controls were antigen-blotted strips probed with rabbit anti-GST.

### Statistical analysis

Unpaired Student *t-*test was used for comparison of the results of the tests and the controls. *P-value* < 0.05 was taken as statistically significant.

## Electronic supplementary material

Supplementary information
