# Publisher Correction: Human single chain-transbodies that bound to domain-I of non-structural protein 5A (NS5A) of hepatitis C virus

**DOI:** 10.1038/s41598-018-24856-4

**Published:** 2018-04-25

**Authors:** Kittirat Glab-ampai, Monrat Chulanetra, Aijaz Ahmad Malik, Thanate Juntadech, Jeeraphong Thanongsaksrikul, Potjanee Srimanote, Kanyarat Thueng-in, Nitat Sookrung, Pongsri Tongtawe, Wanpen Chaicumpa

**Affiliations:** 1 Graduate Program in Immunology, Department of Immunology, Faculty of Medicine Siriraj Hospital, Mahidol University, Bangkok, Thailand; 2 Center of Research Excellence on Therapeutic Proteins and Antibody Engineering, Department of Parasitology, Faculty of Medicine Siriraj Hospital, Mahidol University, Bangkok, Thailand; 3 Graduate Program in Biomedical Science, Faculty of Allied Health Sciences, Thammasat University, Rangsit Campus, Pathum-thani province, 12120 Thailand; 4 School of Pathology, Institute of Medicine, Suranaree University of Technology, Nakhon-ratchaseema province, Thailand

Correction to: *Scientific Reports* 10.1038/s41598-017-14886-9, published online 08 November 2017

The PDF version of this Article contains a typographical error in Table 1 where the last HuscFv clone ‘HuscFv99’ is incorrectly listed as ‘HuscFv5’ in the continued heading. In addition, all the HuscFv clone headings should be shaded. The correct Table 1 appears below.

**Table 1:** Presumptive residues and regions of HCV NS5A bound by the HuscFvs as determined by computerized simulation.

**HCV NS5A protein**		**HuscFv5**		
**Residue**	**Domain**	**Residue(s)**	**Domain**	**Interactive bond**
D18	AH of domain I	R104	VH-CDR3	Salt bridge
L23	AH of domain I	K54	VH-CDR2	H-bond
K26	Domain I	D33	VH-CDR1	Salt bridge
L27	Domain I	S57	VH-CDR2	H-bond
F36	Domain I	Y230	VL-CDR3	π-π interaction
I74	Domain I	F105	VH-CDR3	H-bond
K78	Domain I	S229	VL-CDR3	H-bond
M81	Domain I	Y230	VL-CDR3	H-bond
S229	LCS-I	S204	VL-FR3	H-bond
S232	LCS-I	S204/S190	VL-FR3	H-bond
**HCV NS5A protein**		**HuscFv9**		
**Residues**	**Domain**	**Residue(s)**	**Domain**	**Interactive bonds**
I37	Domain I	R189	VL-FR3	H-bond
T108	Domain I	S165	VL-CDR1	H-bond
E120	Domain I	Y167	VL-CDR1	H-bond
D136	Domain I	N54	VH-CDR2	Salt bridge
P165	Domain I	T102	VH-CDR3	H-bond
K166	Domain I	D31	VH-CDR1	Salt bridge
F169	Domain I	Y33	VH-CDR2	π-π interaction
R170	Domain I	Y167	VL-CDR1	H-bond
D171	Domain I	N59	VH-CDR2	H-bond
E172	Domain I	R65	VH-FR3	Salt bridge
**HCV NS5A protein**		**HuscFv16**		
**Residues**	**Domain**	**Residue(s)**	**Domain**	**Interactive bond**
K107	Domain I	S54	VH-CDR2	H-bond
H124	Domain I	T57	VH-CDR2	H-bond
Y127	Domain I	K224	VL-CDR3	Salt bridge
Y129	Domain I	Y59	VH-CDR2	π-π interaction
E191	Domain I	N225	VL-CDR3	H-bond
R220	Domain I	S30	VH-CDR1	H-bond
E226	Domain I	S30	VH-CDR1	H-bond
D251	Domain II	K224	VL-CDR3	Salt bridge
**HCV NS5A protein**		**HuscFv19**		
**Residues**	**Domain**	**Residue(s)**	**Domain**	**Interactive bond**
H124	Domain I	N31	VH-CDR1	H-bond
G125	Domain I	R100	VH-CDR3	H-bond
Y127	Domain I	Y106	VH-CDR3	H-bond
Y129	Domain I	R98	VH-CDR3	H-bond
S186	Domain I	R59	VH-FR3	H-bond
E191	Domain I	N102	VH-CDR3	H-bond
N248	LCS-I	G26	VH-CDR1	H-bond
T249	LCS-I	R98	VH-CDR3	H-bond
D251	Domain II	R100/N105	VH-CDR3	Salt bridge
**HCV NS5A protein**		**HuscFv99**		
**Residues**	**Domain**	**Residue(s)**	**Domain**	**Interactive bond**
K107	Domain I	T58	VH-CDR2	H-bond
H124	Domain I	K60	VH-FR3	H-bond
G125	Domain I	K60	VH-FR3	H bond
R170	Domain I	D73	VH-FR3	Salt bridge
D171	Domain I	R19	VH-FR1	Salt bridge
E172	Domain I	R19	VH-FR1	Salt bridge
Q187	Domain I	N84/S85	VH-FR3	H-bond
L218/	LCS-I	R57	VH-CDR2	H-bond
G221	LCS-I	R57	VH-CDR2	H-bond
S222	LCS-I	R57	VH-CDR2	H-bond
Y250	Domain II	D62	VH-FR3	H-bond

LCS-1, low complexity sequence between domains I and II of NS5A.

