# Supplementary material for: Human single chain-transbodies that bound to domain-I of non-structural protein 5A (NS5A) of hepatitis C virus
Source: Sci Rep. 2017 Nov 8;7:15042. doi: 10.1038/s41598-017-14886-9 (PMC5678119; doi:10.1038/s41598-017-14886-9)
Supplement: Supplementary file 1 — Supplementary information [file 41598_2017_14886_MOESM1_ESM.pdf]

Human single chain-transbodies that bound to domain-I of non-structural protein 5A (NS5A) of hepatitis C virus

Kittirat Glab-ampai, Monrat Chulanetra, Aijaz Ahmad Malik, Thanate Juntadech, Jeeraphong Thanongsaksrikul, Potjanee Srimanote, Kanyarat Thueng-in, Nitat Sookrung, Pongsri Tongtawe & Wanpen Chaicumpa

Supplementary Table 1. PCR primers used for amplification of DNA sequences coding for NS5A domains I, II, and III

**Primer name**	**Sequence**	**Size of amplicon (bp)**
D1 forward	5’-GAATTCCTCCCCTTCATCTCTTGTCAAAAGG-3’	555
D1 reverse	5’-GTCGACTTAAGTCTCCGCCGTGATGTGGGG-3’	
D2 forward	5’-GAATTCTATGACGTGGACATGGTCGATGCC-3’	280
D2 reverse	5’-GTCGACTTAACAACCAGCAACGGTGGGCGG-3’	
D3 forward	5’-GAATTCAGGAGACGCCGGACAGTGGGT-3’	360
D3 reverse	5’-GTCGACTTAGCAGCACACGGTGGTATCGTC-3’	

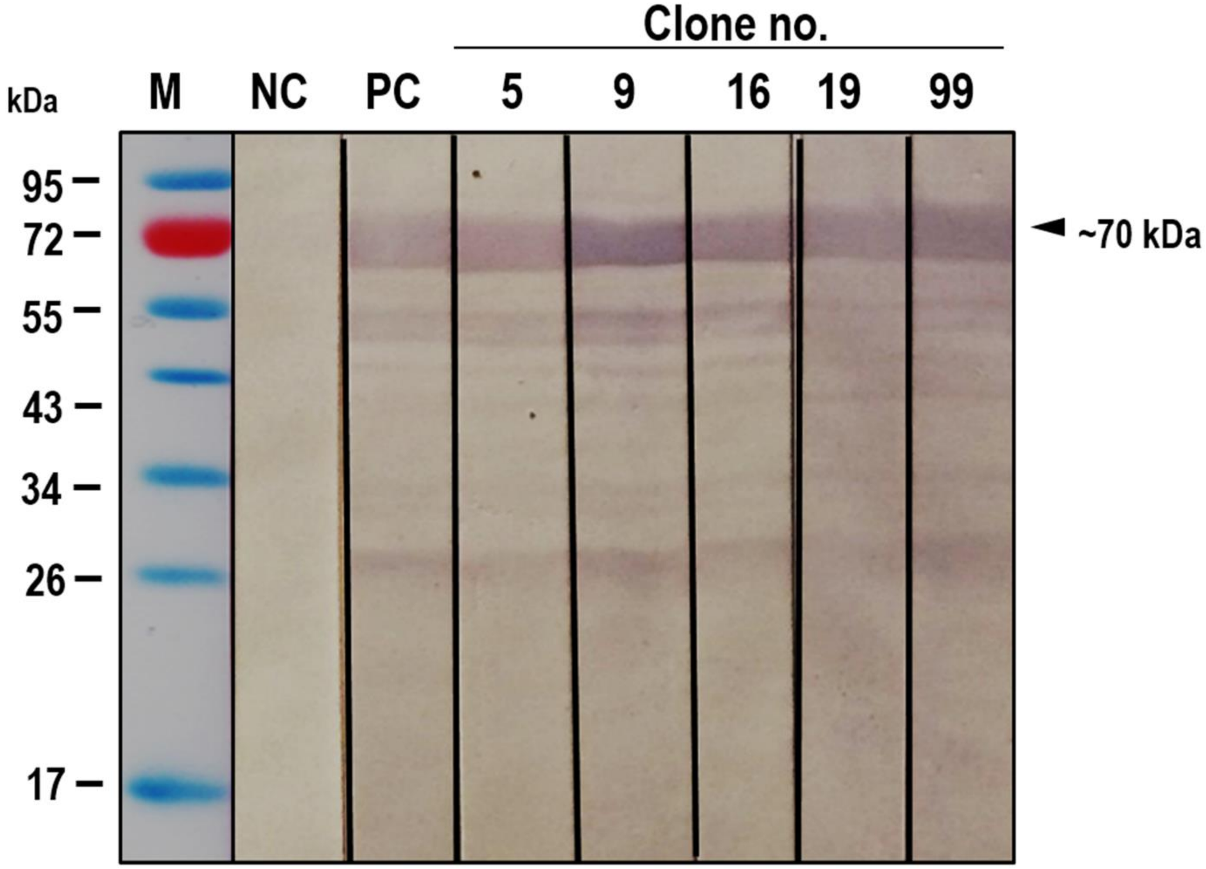

Supplementary Figure S1. Unprocessed immunoblots that relate to Figure 2.

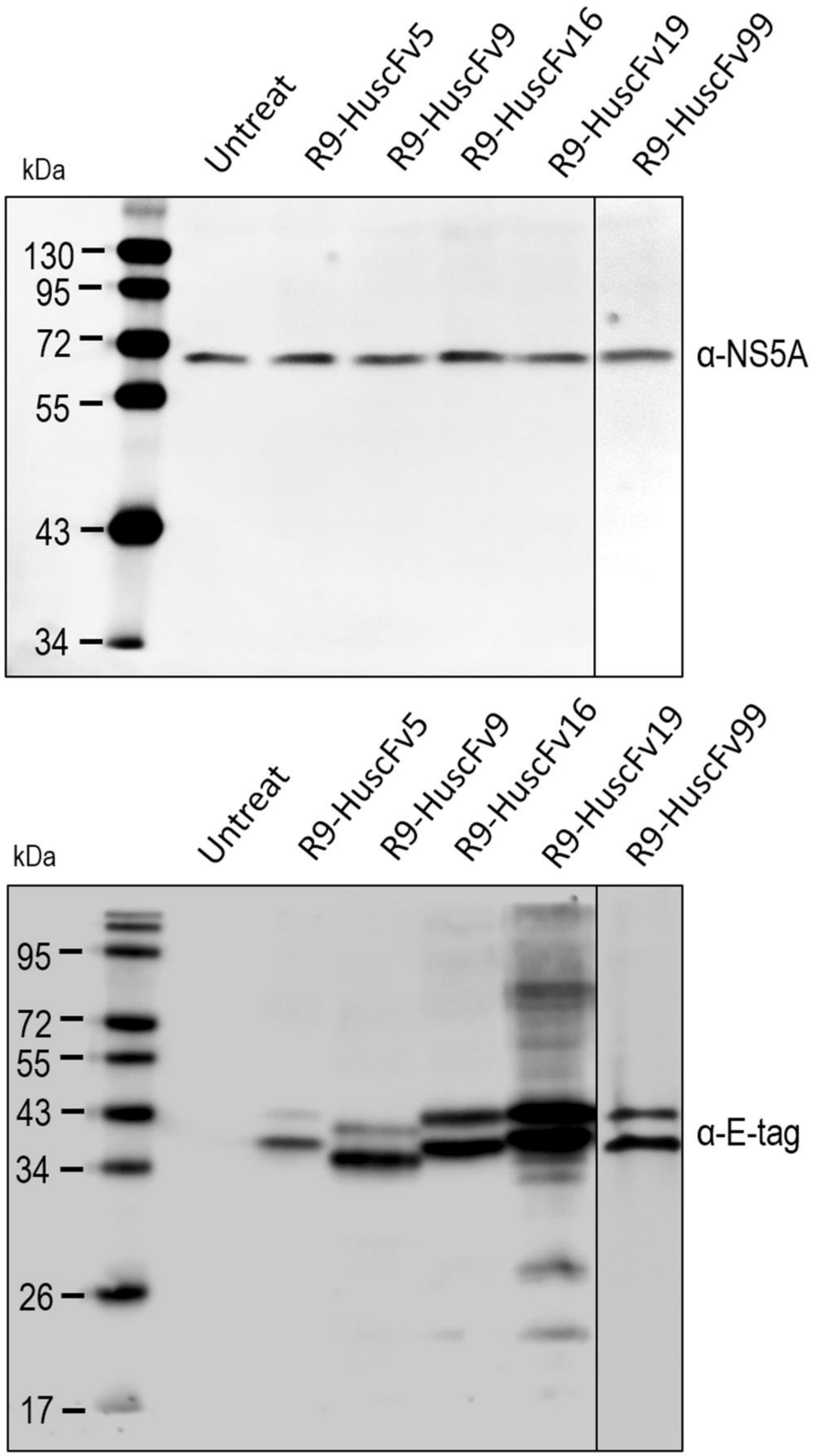

Supplementary Figure S2. Unprocessed immunoblots that relate to Figure 5.

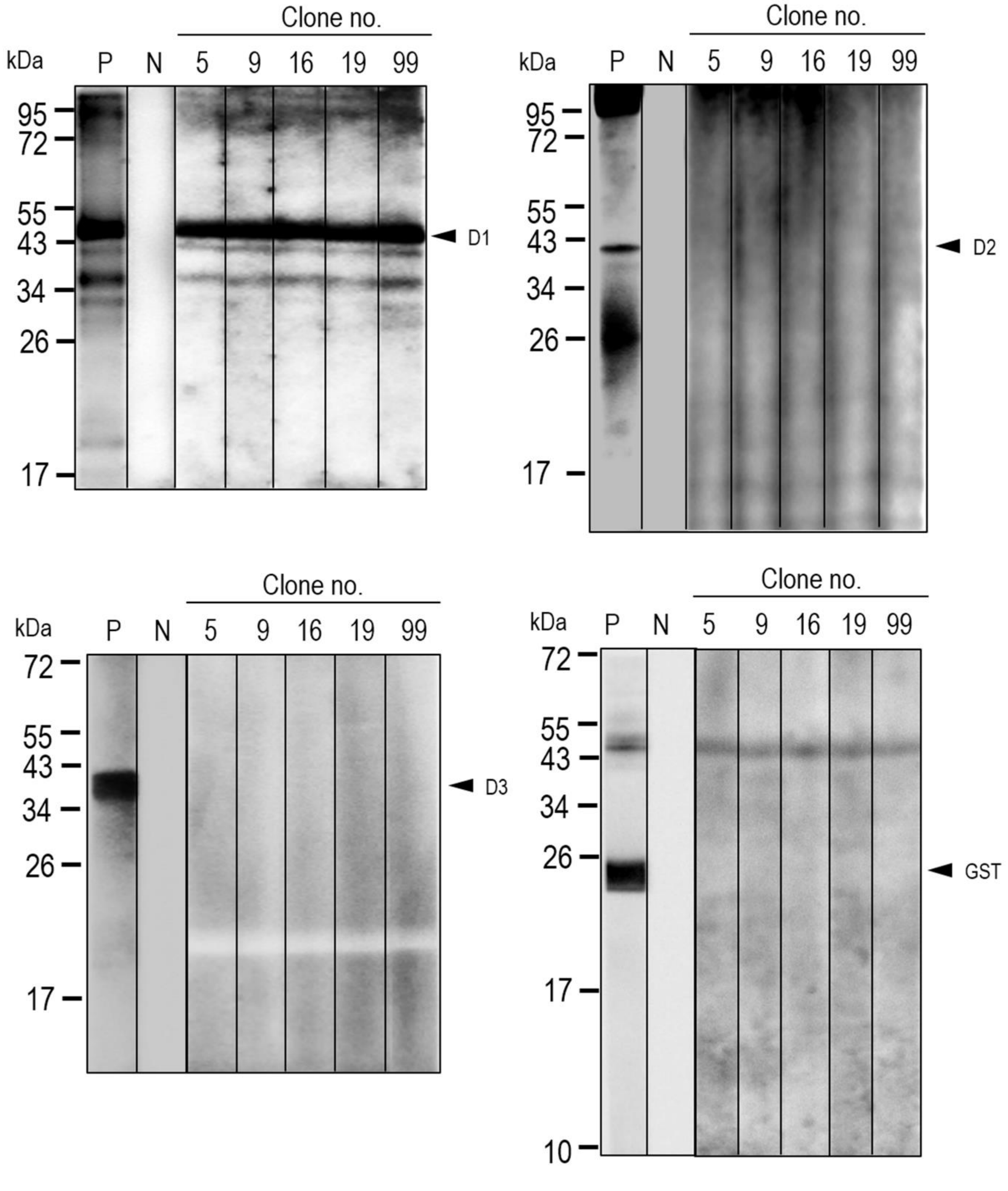

Supplementary Figure S3. Unprocessed immunoblots that relate to Figure 10.
